# Acute Gastritis During Immune Check Point Inhibitor Treatment

**DOI:** 10.1093/jcag/gwab046

**Published:** 2021-12-24

**Authors:** Takahiro Sugawara, Hiroaki Saito, Dai Hirasawa, Jun-ichi Akahira, Akimichi Chonan

**Affiliations:** Department of Gastroenterology, Sendai Kousei Hospital, Sendai, Miyagi, Japan; Department of Gastroenterology, Sendai Kousei Hospital, Sendai, Miyagi, Japan; Department of Gastroenterology, Sendai Kousei Hospital, Sendai, Miyagi, Japan; Department of Pathology, Sendai Kousei Hospital, Sendai, Miyagi, Japan; Department of Internal medicine, Senseki Hospital, Miyagi,Japan

**Figure 1.**
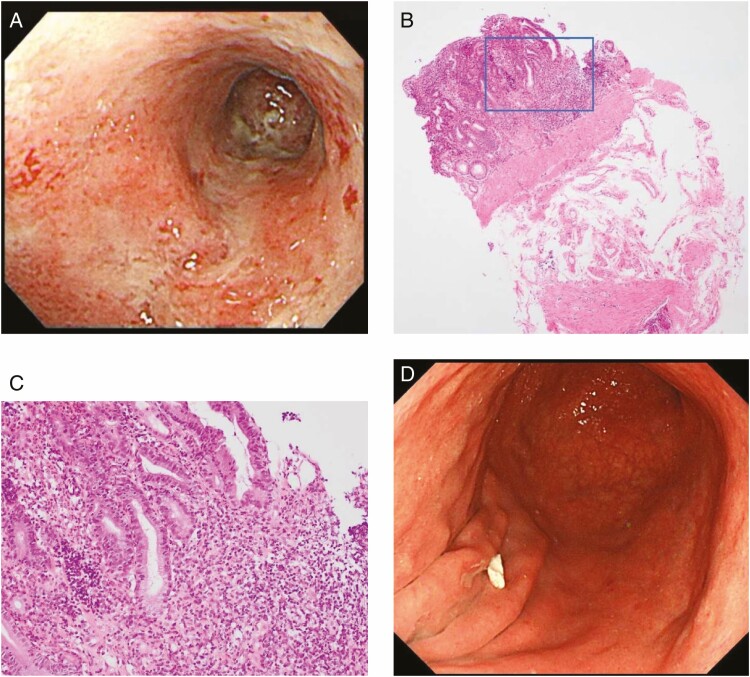
(A) White light endoscopy revealed the diffuse ulcerative changes in the stomach. (B) Biopsy tissue, the gastric mucosa with little normal issue. (C) Inflammatory cells infiltrating the gastric epithelium. (D) Normal gastric mucosal epithelium was observed after 2 months discontinuation of the ICI.

A 73-year-old man with right ureteral cancer was admitted to our hospital after a 10-day history of anorexia and epigastric pain. The symptoms appeared after a 16-cycle dose of pembrolizumab for ureteral cancer. Upper endoscopy revealed diffuse ulcerative changes in the lower part of the stomach with remaining inflammatory mucosa (Figure 1A). Histopathological examination showed numerous inflammatory cells, mainly lymphocytes, infiltrating the gastric epithelium, and apoptotic changes (Figure 1B,C). Helicobacter pylori was not found. Considering the possibility of gastritis caused by immune checkpoint inhibitors (ICIs), pembrolizumab  administration was discontinued. After 2 months, upper endoscopy revealed that the inflammatory changes in the gastric mucosa had improved (Figure 1D), and the subjective symptoms had disappeared completely.

Gastritis has been reported as a rare adverse event of ICIs. The pathological characteristics of ICI-related gastritis remain unclear. Inflammatory cell infiltration, mainly of lymphocytes, and the presence of apoptotic bodies are considered characteristics of ICI-related gastritis; these were observed in the present case. Cases that do not respond to supportive treatment require corticosteroid therapy.

